# Elevated pulsatility index of the superior mesenteric artery indicated prolonged mechanical ventilation in patients after cardiac valve surgery

**DOI:** 10.3389/fsurg.2022.1049753

**Published:** 2023-01-06

**Authors:** Yuankai Zhou, Huaiwu He, Na Cui, Xiaoting Wang, Yun Long, Dawei Liu

**Affiliations:** Department of Critical Care Medicine, State Key Laboratory of Complex Severe and Rare Diseases, Peking Union Medical College Hospital, Peking Union Medical College, Chinese Academy of Medical Sciences, Beijing, China

**Keywords:** pulsatility index of the superior mesenteric artery, prolonged mechanical ventilation, cardiac valve surgery, intestinal perfusion, Doppler ultrasound (DUS)

## Abstract

**Purpose:**

This study examined whether alterations in Doppler parameters of superior mesenteric artery (SMA) are associated with prolonged mechanical ventilation (PMV) in patients who underwent cardiac valve surgery.

**Methods:**

Hemodynamic and SMA Doppler parameters were collected at intensive care unit(ICU) admission. The duration of mechanical ventilation was monitored. PMV was defined as mechanical ventilation ≥96 h.

**Results:**

A total of 132 patients admitted to ICU after cardiac valve surgery were evaluated for enrollment, of whom 105 were included. Patients were assigned to the control (*n* = 63) and PMV (*n* = 42) groups according to the mechanical ventilation duration. The pulsatility index(SMA-PI) and resistive index of SMA (SMA-RI) were 3.97 ± 0.77 and 0.88 (0.84–0.90) in the PMV group after cardiac valve surgery, which was lower than the SMA-PI (2.95 ± 0.71, *p *< 0.0001) and SMA-RI of controls (0.8, 0.77–0.88, *p *< 0.0001). SMA-PI at admission had favorable prognostic significance for PMV (AUC = 0.837, *p* < 0.0001).

**Conclusions:**

An elevated SMA-PI is common in patients after cardiac valve surgery with PMV. Increased SMA-PI could help predict PMV after cardiac valve surgery. Using point-of-care ultrasound to measure SMA-PI at ICU admission is an acceptable and reproducible method for identifying patients with PMV.

## Introduction

Cardiac surgery causes tissue hypoperfusion and an excessive inflammatory response, leading multiple organ failure, including that of the gastrointestinal tract. Despite advances in surgical methods and perioperative intensive care management, there is no consensus on the role of gastrointestinal perfusion in the resuscitation of patients who underwent cardiac surgery.

The intestinal blood flow self-regulation function is weak, which was different from brain, kidney and skeletal muscle (1). Cardiopulmonary bypass induced severe stress, cardiogenic shock, and use of vasopressor could worsen the intestinal perfusion after cardiac surgery. However, it should be noticed that the intestine is not only a digestive organ but also an immune organ. Intestine is considered to be the important bacterial barrier and “the initiator of sepsis in critical care status (2). Intestinal bacterial translocation and cytokine release caused by intestinal ischemia/reperfusion might cause the intestine original ARDS. Then, these may lead to prolonged mechanical ventilation (PMV).

Therefore, monitoring arterial blood flow in the intestine may be a potential method for assessing intestinal resuscitation status. The small intestine receives the most blood through the superior mesenteric artery (SMA). The SMA resistance index (SMA-RI) calculated by Doppler ultrasound can indicate the resistance of the whole distal intestinal circulation (3, 4), since splanchnic hypoperfusion alters organ microcirculation by increasing the flow resistance (5, 6). In patients who underwent cardiac surgery, elevated SMA-RI is related to lactate levels and kinetics (7).

PMV is related to a high hospital mortality rate (13%–50%), a longer intensive care unit (ICU) stay, and significantly poorer quality of life in patients after cardiac surgery (8, 9). Because this matches the International Classification of Diseases (ICD) code 96.72 used in many large-scale studies (10), PMV is usually recognized as a definition of ≥96 h. This study aimed to verify whether the high-resistance state of the intestinal perfusion arteries predicts PMV. This may help physicians screen out the shock state with hypoperfusion of visceral organs, which may lead to PMV.

## Material and methods

### Patient enrolment

This prospective observational study was approved by the Institutional Research and Ethics Committee of Peking Union Medical College Hospital for human subjects. Before enrollment, written informed consent was obtained from all patients or their next of kin.

The trial was performed in a 15-bed adult critical care unit from January 2021 to March 2022.

The inclusion criteria: (1) patients admitted to the ICU immediately after cardiac valve surgery, (2) age 18–80 years, (3) sinus rhythm, (4) no active bleeding or pneumothorax.

The exclusion criteria: (1) history of peripheral vascular disease; (2) severe stenosis defined as SMA peak systolic velocity (SMA-PSV) / abdominal aorta peak velocity >3 or SMA-PSV >275 cm/s (11); (3) end-stage renal disease or advanced cancer; (4) liver cirrhosis, portal hypertension or hepatic dysfunction; and (5) poor quality of abdominal ultrasound images.

### Doppler ultrasound monitoring

Doppler ultrasound was performed with an ultrasound system consisting of a C60xp probe with 2–5 MHz (X-Porte Ultrasound System, SONOSITE, INC., United States). Doppler parameters were measured by two physicians; each physician performed two assessments and averaged the results of the two measurements. Two ICU physicians had more than 4 years of experience in critical ultrasound and obtained certification from the Chinese Critical Ultrasound Study Group. Two skilled physicians conducted 40 measurements on ten patients after cardiac valve surgery to evaluate inter-observer reproducibility.

SMA Doppler parameters were measured 10 mm proximal to the abdominal aorta. The angle of insonation was <60°. The time-velocity waveform readings were used to calculate the SMA end-diastolic velocity (EDV), peak systolic velocity (PSV), resistance index (RI), time-averaged mean velocity (TAMV), and pulsatility index (PI), according to the calculation software carried by the ultrasound. PI = (PSV - EDV)/TAMV, and RI = (PSV - EDV)/PSV.

### Data collection

Demographic characteristics were determined for further analyses (e.g., sex, age, EuroSCORE-II, body mass index (BMI), sequential organ failure assessment (SOFA) score, hemodynamic parameters, and blood gas analysis). In addition, the types of operations and operation times were collected.

The vasopressor–inotrope score (VIS) was used to evaluate the vasopressor and inotrope dosages. The VIS was calculated as follows: VIS = dobutamine dose (µg/kg/min) + dopamine dose (µg/kg/min) + [10,000 * vasopressin dose (U/kg/min)] + [10 * milrinone dose (µg/kg/min)] + [100 * epinephrine dose(µg/kg/min)] + [100 * norepinephrine dose (µg/kg/min)]. Moreover, prognostic indicators (e.g., duration of MV, hospital mortality, and length of ICU stay) were recorded.

### Statistical analysis

The mean ± standard deviation or median and interquartile range (IQR) were used to show demographic information. The Shapiro-Wilk test was used to assess the normality of continuous numerical data. Student's t-test or Mann-Whitney U-test were used to compare the differences between-groups for continuous variables. Categorical variables were compared between the patient groups using the Chi-square test. Correlations between PMV and variables were calculated *via* logistic regression analysis. Variables that showed a statistically significant effect in the univariate logistic regression were selected and included in the multivariate logistic regression. Furthermore, receiver operating characteristic (ROC) curves were constructed to evaluate the predictability of these variables for PMV. Statistical significance was set at *p* < 0.05. All analyzed *p*-values were two-sided. Statistical analyses were performed using SPSS software (version 25.0; SPSS, Chicago). Bland–Altman plots with the mean difference and 95 percent limit of agreement (LOA) were performed using MedCalc Statistical software (version 19.7; Ostend, Belgium) and used to assess inter-observer agreement, and statistical analyses.

## Results

### General characteristics

**Figure 1:**
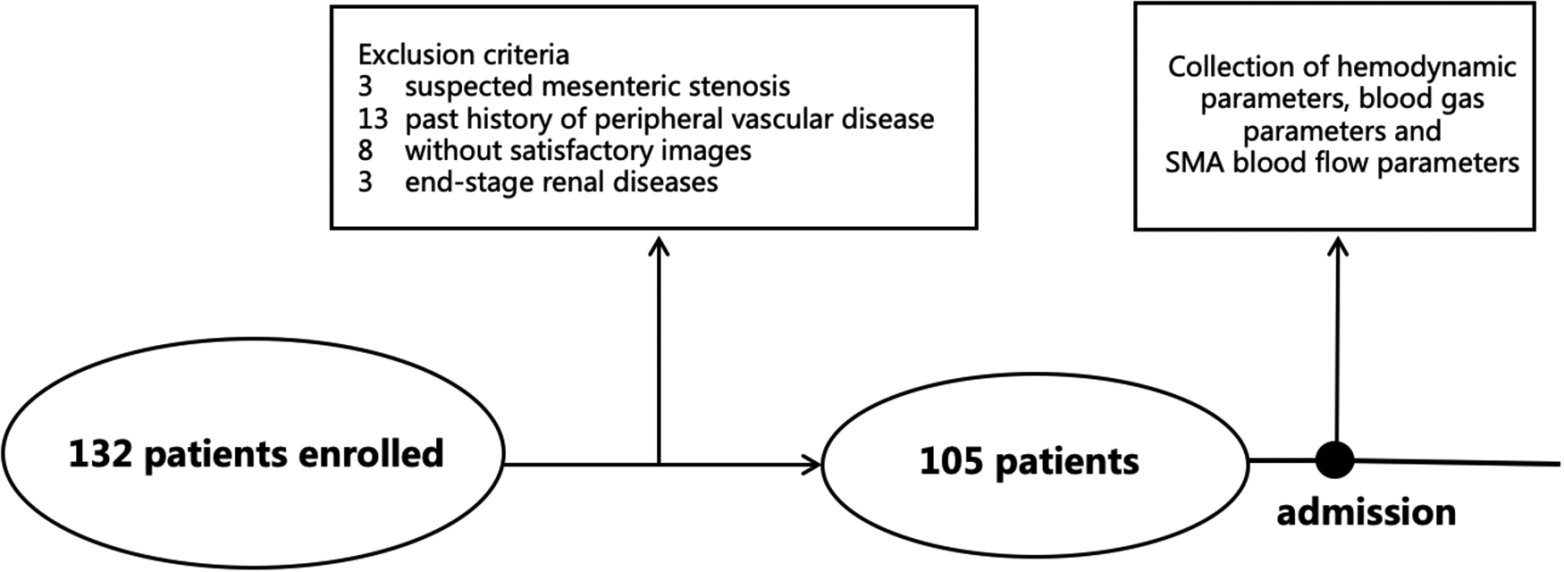
Protocol of the study. SMA, superior mesenteric artery.

In total, 132 patients were enrolled after cardiac valve surgery, and 105 cases were included in the final analysis (Figure 1). Of the 27 ineligible patients, 13 were excluded because of history of peripheral vascular disease, eight because of unsatisfactory images, three because of suspected mesenteric stenosis, and three because of end-stage renal disease. Of the 105 patients included, 63 had less than 96 h of mechanical ventilation (control group) and 42 had 96 h or more (PMV group). The demographic characteristics are shown in Table 1.

**Table 1:** Baseline characteristics of the patients after cardiac valve surgery.

	Total (*n* = 105)	Control group (*n* = 63)	PMV group (*n* = 42)	*p-*value
Age, y	58 (49–66)	52 (47–62)	65 (57–75)	<0.0001
BMI, kg/m^2^	23.3 ± 2.1	23.6 ± 2.0	23.0 ± 2.1	0.164
SOFA score	8 (7–9)	8 (7–9)	9 (8–11)	<0.0001
EuroSCORE II, (%)	1.45 (0.97–3.06)	1.07 (0.75–1.97)	2.86 (1.32–5.89)	<0.0001
NYHA, *n* (%)				
I,II	74 (70.5)	55 (87.3)	19 (45.2)	<0.0001
III,IV	31 (29.5)	8 (12.7)	23 (54.8)	<0.0001
pre-LVEF, %	52.4 ± 10.5	56.9 ± 8.54	45.7 ± 9.47	<0.0001
pre-cardiac surgery, *n* (%)	2 (2)	0 (0)	2 (5)	0.31
CPB time, min	95 (86–111)	89 (84–98)	113 (98–126)	<0.0001
E at admission, ug/kg/min	0.05 (0.02–0.08)	0.03 (0.00–0.05)	0.08 (0.05–0.10)	<0.0001
NE at admission, ug/kg/min	0.18 (0.10–0.32)	0.13 (0.08–0.24)	0.24 (0.17–0.42)	0.0003
Cr, µmoI/L	79 (61–95)	68 (52–83)	94 (82–169)	<0.0001
Type of operation, *n* (%)				
Isolated valve surgery	62 (59.1)	50 (79.4)	12 (28.6)	<0.0001
Multiple valve surgery	43 (40.9)	13 (20.6)	30 (71.4)	<0.0001
Co-morbidities, *n* (%)				
COPD	28 (26.7)	16 (25.4)	12 (27.9)	0.72
IE	36 (34.3)	25 (39.7)	11 (26.2)	0.22
Hypertension	27 (25.7)	15 (23.8)	12 (28.6)	0.58
Diabetes mellitus	12 (11.4)	7 (11.1)	5 (11.9)	0.93
Stroke	4 (3.8)	2 (1.6)	2 (4.8)	0.68
Length of ICU stay, d	6 (3–8)	4 (3–5)	9 (7–11)	<0.0001
Time of MV, h	78 (39–133)	46 (29–70)	144 (116–203)	<0.0001
Mortality, n (%)	3 (2.9)	0 (0.0)	3 (7.1)	0.04

Values are given as mean + SD or median (interquartile range).

MV, Mechanical ventilation; BMI, Body mass index; SOFA, Sequential organ failure assessment; NYHA, New York Heart Association classification; CPB, Cardiopulmonary bypass; E, Epinephrine; NE, Norepinephrine; IE, Infective endocarditis; COPD, Chronic obstructive pulmonary disease; ICU, Intensive care unit; EF, Left ventricular ejection fraction.

### Consistency of measurement

**Figure 2:**
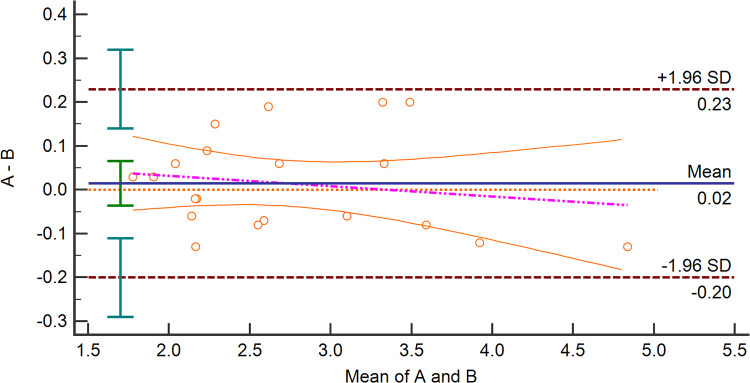
Bland–Altman analysis plots about Inter-Observers agreement for measuring SMA-PI. SMA-PI, the pulsatility index of the superior mesenteric artery.

Figure 2 presents the Bland–Altman analysis plots of the inter-observer agreement for the SMA-PI measurements of 20 patients after cardiac valve surgery. The mean bias of the two operators was 0.02 (LOA: −0.20, 0.23), and there was no significant difference between the measurements of the two skilled physicians (*p* = 0.55).

### Parameters at admission to the intensive care unit

The hemodynamic and Doppler parameters of the patients upon admission to the ICU after cardiac valve surgery are listed in Table 2. There were no statistically significant differences between the heart rate (HR), mean arterial pressure, central venous pressure (CVP), or other basic hemodynamic parameters in the control and PMV groups. In the PMV group, the SMA-PI, SMA-RI, and lactate values were significantly higher than in the control group (*p* < 0.0001).

**Table 2:** The hemodynamic and Doppler variables at admission of the patients after cardiac valve surgery.

Variable	Total (*n* = 105)	Control group (*n* = 63)	PMV group (*n* = 42)	*p-*value
HR (bpm)	92 ± 12	91 ± 11	95 ± 9	0.07
MAP (mmHg)	80 ± 7.0	81 ± 6	79 ± 8	0.13
CVP (mmHg)	8[7–10]	8[7–9]	9[7–10]	0.06
PaO_2_ (mmHg)	98[84–116]	94[84–111]	101[86–126]	0.21
Pv-aCO_2_ (mmHg)	5.0 ± 1.9	4.5 ± 1.7	5.6 ± 2.1	0.005
ScvO_2_ (%)	70.4 ± 7.0	71.3 ± 5.7	69.2 ± 8.5	0.14
Lactate (mmol/L)	5.1 ± 2.3	4.2 ± 1.5	6.4 ± 2.7	<0.0001
HGB (g/L)	111[98–119]	108[95–118]	112[101–120]	0.26
SMA-PI	3.35 ± 0.89	2.95 ± 0.71	3.97 ± 0.77	<0.0001
SMA-RI	0.86[0.80–0.89]	0.82[0.77–0.88]	0.88[0.84–0.90]	<0.0001

Values are given as mean + SD or median (interquartile range).

PMV, prolonged mechanical ventilation; MAP, mean arterial pressure; CVP, central venous pressure; PaO_2_, partial pressure of oxygen; ScvO_2_, superior vena cava oxygen saturation; Pv-aCO_2_, arterial-venous carbon dioxide partial pressure difference; HGB, hemoglobin; SMA-PI, the pulsatility index of the superior mesenteric artery; SMA-RI, the resistive index of the superior mesenteric artery.

Then, the prognostic significance of the relevant variable for PMV was distinguished by logistic regression analysis. Univariate logistic regression indicated the correlations between PMV and SMA-PI (odds Ratio [OR] per 10^−1^ units: 5.594, 95% confidence interval [CI]: 2.296–13.631, *p *< 0.0001) and SMA-RI (OR per 10^−1^ units: 1.188, 95% CI: 1.087–1.299, *p *< 0.0001).At the same time, PMV was also related to patient age, cardiopulmonary bypass(CPB) time, creatinine and left ventricular ejection fraction(EF) before surgery, lactate value, arterial-venous carbon dioxide partial pressure difference(Pv-aCO_2_), norepinephrine(NE) dose, CVP, and HR at admission (Table 3).

**Table 3:** Logistic regression for baseline characteristics and variables at admission to predict prolonged mechanical ventilation.

Variable	Univariate analysis			Multivariate analysis		
	OR	95% CI	*p*-value	OR	95% CI	*p*-value
Age	1.075	1.035–1.117	<0.0001	1.049	0.991–1.111	0.099
Gender	1.776	0.793–3.979	0.163			
BMI	0.872	0.718–1.058	0.164			
CPB time	1.087	1.050–1.125	<0.0001	1.053	0.998–1.111	0.060
Pre-operation						
Creatinine	1.024	1.011–1.038	<0.0001	1.010	0.997–1.024	0.133
EF	0.874	0.827–0.924	<0.0001	0.935	0.859–1.017	0.115
ICU admission after operation						
SMA-PI (OR per 10^−1^ units)	5.594	2.296–13.631	<0.0001	1.182	1.032–1.355	0.016
SMA-RI (OR per 10^−2^ units)	1.188	1.087–1.299	<0.0001	0.972	0.821–1.152	0.745
Lactate	1.696	1.318–2.182	<0.0001	0.765	0.459–1.274	0.303
MAP	0.956	0.902–1.013	0.127			
NE (OR per 10^−2^ units)	1.562	1.308–1.865	<0.0001	1.310	1.006–1.707	0.045
ScvO_2_	0.957	0.903–1.015	0.142			
Pv-aCO_2_	1.364	0.901–1.709	0.07			
CVP	1.220	1.011–1.473	0.038	1.056	0.769–1.450	0.738
HR	1.043	1.005–1.082	0.027	0.979	0.929–1.041	0.494

BMI, body mass index; CPB, cardiopulmonary bypass; EF, Left ventricular ejection fraction before surgery; SMA-PI, the pulsatility index of the superior mesenteric artery; SMA-RI, the resistive index of the superior mesenteric artery; MAP, mean arterial pressure; NE Norepinephrine; ScvO_2_,superior vena cava oxygen saturation; Pv-aCO_2_,arterial-venous carbon dioxide partial pressure difference; CVP, central venous pressure; HR, heart rate; OR, Odds Ratio; CI, confidence interval.

**Figure 3:**
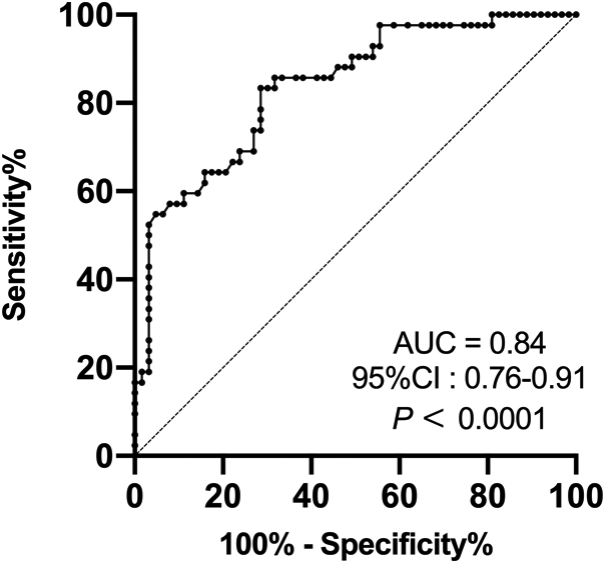
Receiver operating characteristic curve of SMA-PI's ability to predict PMV in patient after cardiac valve surgery. SMA-PI, the pulsatility index of the superior mesenteric artery; PMV, prolonged mechanical ventilation; AUC, area under curve; CI, confidence interval.

According to a thorough multiple stepwise logistic regression analysis using the factors mentioned above, the significant associations between SMA-PI (OR, 1.182; 95% CI: 1.032–1.355, *p *= 0.016), NE dose at admission (OR, 1.310; 95% CI: 1.006–1.707, *p *= 0.045), and PMV were discovered (Table 3). SMA-PI also showed a strong predictive value for PMV, with an area under the ROC curve of 0.84 (95% CI: 0.76–0.91, *p *< 0.0001, Figure 3). With a cut-off value of 3.31, SMA-PI had a sensitivity of 83.33% and a specificity of 71.43% for PMV, respectively.

## Discussion

To our knowledge, this is the first study that explored the relationship between gastrointestinal perfusion blood flow and PMV in patient post-operation of cardiac valve surgery.

This study had two main findings. First, we found that patients with PMV after cardiac valve surgery had a markedly elevated SMA-PI at ICU admission. Second, SMA-PI effectively predicted PMV in patients after cardiac valve surgery.

After cardiac surgery, patients are usually weaned from mechanical ventilation once they recover from anesthesia. However, some patients experience cardiogenic shock or severe stress caused by cardiopulmonary bypass. These factors could reduce perfusion to the intestine, and lead to intestinal ischemia. At the same time, these factors are also the main reasons for prolonging the duration of mechanical ventilation. Intestinal ischemia might weaken its barrier function and lead to sepsis of intestinal origin1, ultimately worsening patient prognosis. Therefore, monitoring blood flow in the intestine is relevant in critically ill patients (12, 13). PI and RI are indicators that can respond to blood flow and microcirculatory resistance in the distal part of the vessel (3, 4). The correlation between the SMA-RI and lactate levels and kinetics in post-cardiac surgery patients was identified in our previous study.

In the present study, SMA-PI and RI values in the PMV group were significantly higher than those in the control group. This suggests that decreased blood flow and impaired microcirculation of the intestine are associated with PMV in patients after cardiac valve surgery.

Univariate regression analysis results were similar to previous studies which examined the risk factors for PMV after cardiac surgery, i.e., age, CPB time (14), preoperative creatinine, preoperative EF (15), lactate at admission and a high dose of vasopressors (16).

**Figure 4:**
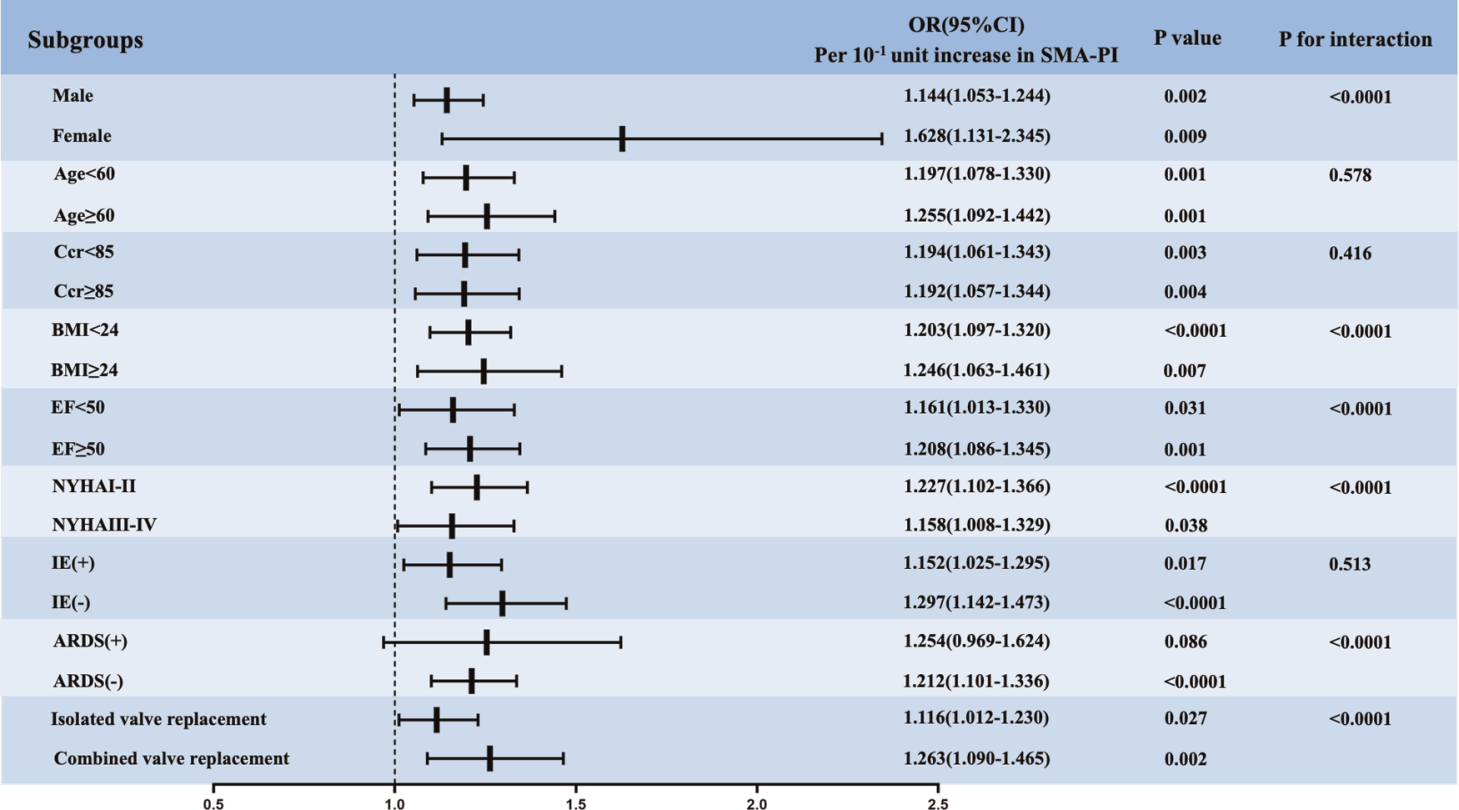
Logistic regression analysis evaluating the implication of SMA-PI in predicting PMV in subgroups. SMA-PI, the pulsatility index of the superior mesenteric artery; PMV, prolonged mechanical ventilation; Ccr, endogenous creatinine clearance rate; BMI, body mass index; EF, left ventricular ejection fraction before surgery; NYHA, New York heart association; IE, infective endocarditis; ARDS, acute respiratory distress syndrome; OR, Odds ratio; CI, confidence interval.

We also acknowledge that many factors besides shock and severe stress can affect the PI and RI values of the SMA, such as age, sex, chronic diseases such as hypertension and diabetes mellitus, and the use of vasopressors. Therefore, we chose non-coronary artery bypass grafting to reduce the impact of underlying peripheral vascular disease. A subgroup comparison was also conducted, and the results showed that SMA-PI could predict PMV independent of age and sex (Figure 4). Moreover, many factors that could affect the duration of mechanical ventilation were analyzed by subgroup comparisons (Figure 4). The results showed that the SMA-PI could not predict PMV in patients with ARDS after cardiac valve surgery. ARDS has an independent and important effect on weaning from mechanical ventilation.

In models with multivariate stepwise logistic regression, SMA-PI and NE dose still predicted PMV (Table 3). NE dose is not only related to the severity of shock but also affects intestinal vascular resistance, which in turn affects SMA-PI. This result reinforces the need for SMA blood flow monitoring and the need to pay attention to the negative effects of vasopressors on intestinal blood flow (12). Recent studies on critically ill patients with non-obstructive mesenteric ischemia showed that the vasopressor dose affects the diastolic effect of prostacyclin E1 on the SMA (17).

This is precisely because blood flow in the SMA is the result of the influence of many factors, and blood flow monitoring of the SMA is of greater significance in patients after cardiac surgery. However, we also have multiple therapeutic approaches that can improve blood flow when altered blood flow in the SMA is detected. The key to achieving this purpose might be the real-time and bedside monitoring of SMA blood flow using Doppler.

The intestine is an important immune and digestive organ that is particularly prone to a decrease in blood flow in the critical care state. Monitoring and intervention for intestinal hypoperfusion may be a potential way to reduce the inflammatory response, reduce organ dysfunction due to perioperative shock, and improve prognosis.

This research had some limitations: (1) The SMA-PI in basic state before surgery was not measured, because water fasting measures were required only for 6 h before surgery, and the interference of feeding on SMA blood flow could not be removed. However, patients with coronary heart disease and peripheral vascular disease, which were used to reduce the influence of vascular disease on SMA blood flow, were excluded. (2) SMA-PI can only partially reflect the blood flow status of the SMA. (3) SMA-PI could be influenced by many factors, such as cardiac output, vasopressors, intra-abdominal pressure and vascular elasticity, the non-obstructive intestinal ischemia seen in a few post-cardiac surgery patients is due to the cumulative effect of various factors on the intestinal circulation. Therefore, we evaluated the consequences of these factors. (4) The SMA-PI monitoring was not dynamic.

In conclusion, an elevated SMA-PI is common and is associated with PMV in patients after cardiac valve surgery. Increased SMA-PI could act as a predictor of PMV in these patients. Using point-of-care ultrasound to assess SMA-PI at ICU admission is an acceptable and reproducible method for identifying PMV patients after cardiac valve surgery.

## Data Availability

The raw data supporting the conclusions of this article will be made available by the authors, without undue reservation.
